# Intermittent Fever

**Published:** 1899-03

**Authors:** J. A. Whitman

**Affiliations:** Beaufort, S. C.


### INTERMITTENT FEVER.

J. A. Whitman, M. D., Beaufort, S. C.

While visiting in the North the past summer I met with a case of chills and fever that would not yield to Quinine. A young girl 17 years old was taken the first of June with her first menstrual period, which was accompanied with intermittent fever occurring every other day. A druggist treated her with Quinine until he was satisfied that Quinine would not cure it. I saw the case the first of September, and took such symptoms as I could get, and looked them up and decided that Sulphur was the remedy. Not feeling so certain of my remedy I consulted an old friend who had been practicing Homœopathy forty years. After reading my notes to him he suggested Pulsatilla, but upon studying that remedy it did not correspond with my notes. I asked him to look at Sulphur; upon doing so he concluded Sulphur might be the simillimum. I went to the patient and gave her five drops of Sulphur, Mx potency. On leaving, she wanted to know if I was not going to leave any medicine for her. I informed her no; only what I had put on her tongue. She and her friends thought it strange that that dose would cure her of an ague of three months' duration. I told them we would wait and see. Her next chill day I met the druggist and asked about the chill; he informed me she missed it, and he wanted to know of me if I really thought that one dose of so highly a diluted drug would cure the case, I told him yes, or I would not have given it, and farther, that her periods, which had been suspended during the time would re-appear. He said that was too much for him. I told him that was the trouble with the most of the homœopathists in the vicinity. I did not hear anything from the case for about four weeks, when I called on him and asked about the girl. He told me about seven days after the last chill she had another; that was the last, and not long after that her periods came on, and she was all right; but he could not comprehend it; it was too much for him.

